# Correlation of Mycobacterium Tuberculosis Specific and Non-Specific Quantitative Th1 T-Cell Responses with Bacillary Load in a High Burden Setting

**DOI:** 10.1371/journal.pone.0037436

**Published:** 2012-05-22

**Authors:** Grant Theron, Jonny Peter, Laura Lenders, Richard van Zyl-Smit, Richard Meldau, Ureshnie Govender, Keertan Dheda

**Affiliations:** 1 Lung Infection and Immunity Unit, Division of Pulmonology and UCT Lung Institute, Department of Medicine, University of Cape Town, Cape Town, South Africa; 2 Institute of Infectious Diseases and Molecular Medicine, University of Cape Town, Cape Town, South Africa; National Institute for Infectious Diseases (L. Spallanzani), Italy

## Abstract

**Background:**

Measures of bacillary load in patients with tuberculosis (TB) may be useful for predicting and monitoring response to treatment. The relationship between quantitative T-cell responses and mycobacterial load remains unclear. We hypothesised that, in a HIV-prevalent high burden setting, the magnitude of mycobacterial antigen-specific and non-specific T-cell IFN-γ responses would correlate with (a) bacterial load and (b) culture conversion in patients undergoing treatment.

**Methods:**

We compared baseline (n = 147), 2 (n = 35) and 6 month (n = 13) purified-protein-derivative (PPD) and RD1-specific (TSPOT.TB and QFT-GIT) blood RD1-specific (TSPOT.TB; QFT-GIT) responses with associates of sputum bacillary load in patients with culture-confirmed TB in Cape Town, South Africa.

**Results:**

IFN-γ responses were not associated with liquid culture time-to-positivity, smear-grade, Xpert MTB/RIF-generated cycle threshold values or the presence of cavities on the chest radiograph in patients with culture-confirmed TB and irrespective of HIV-status. 2-month IGRA conversion rates (positive-to-negative) were negligible [<11% for TSPOT.TB (3/28) and QFT-GIT (1/29)] and lower compared to culture [60% (21/35); p<0.01].

**Conclusions:**

In a high burden HIV-prevalent setting T-cell IFN-γ responses to *M. tuberculosis-*specific and non-specific antigens do not correlate with bacillary load, including Xpert MTB/RIF-generated C_T_ values, and are therefore poorly suited for monitoring treatment and prognostication.

## Introduction

Measures of bacillary load at diagnosis or after the intensive phase of anti-TB treatment are crudely associated with relapse and treatment failure in tuberculosis (TB) patients, yet better and more accurate prognostic and treatment monitoring tools are needed [1]. Such tools may identify high risk patients and facilitate the more rapid evaluation of new therapeutic interventions for TB. It has been suggested that immunodiagnostic biomarkers such as interferon-γ (IFN-γ) release assays (IGRAs) may serve as surrogate markers of bacillary load and treatment response [2]. Although the diagnostic accuracy of standardised IGRAs [TSPOT.TB (Oxford Immunotec, UK) and QuantiFERON TB Gold-in-tube (QFT-GIT; Cellestis, Australia)] for latent and active TB have been extensively studied [3]–[9], there are hardly any data about their relationship with bacterial load [10]. The relationship between bacillary burden and the magnitude of IFN-γ in response to RD-1 specific antigens thus remains uncertain [11]. Their role as tools of prognostication and disease monitoring have also been little studied, particularly in high burden settings.

A previous study performed in a small number of HIV-infected TB patients demonstrated a crude relationship between antigen-specific T-cell responses and sputum bacillary load [10]. This finding is supported by the longitudinal increase in quantitative IFN-γ responses in TB contacts prior to diagnosis with active TB [12], [13], as well as declining IFN-γ responses seen in TB patients in low burden settings undergoing treatment [14]–[16]. This latter aspect remains inconsistent, however, as several studies have reported persistently increased positive responses during and after treatment [17]–[25]. Thus, the role of IGRAs as tools of prognostication and disease monitoring continues to remain unclear, especially with regard to their utility in high burden settings. Evaluating the relationship between quantitative T-cell responses using both standardised IGRAs (TSPOT.TB and QFT-GIT) and bacillary load at diagnosis or during treatment has hitherto not been undertaken. These aspects are also largely unexplored with regard to non-specific antigens such as *M. tuberculosis*-derived purified protein derivative (PPD) and newer measures of bacillary load [such as the World Health Organisation-recommended Xpert MTB/RIF assay [26]].

To address these gaps in our knowledge we comparatively evaluated the magnitude of Th1-based *M. tuberculosis*-specific and non-specific quantitative T cell responses with measures of sputum bacillary load, some of which are known to be associated with treatment outcomes, prior to and during treatment in TB patients from Cape Town, South Africa.

## Materials and Methods

### Study Population

**Figure 1:**
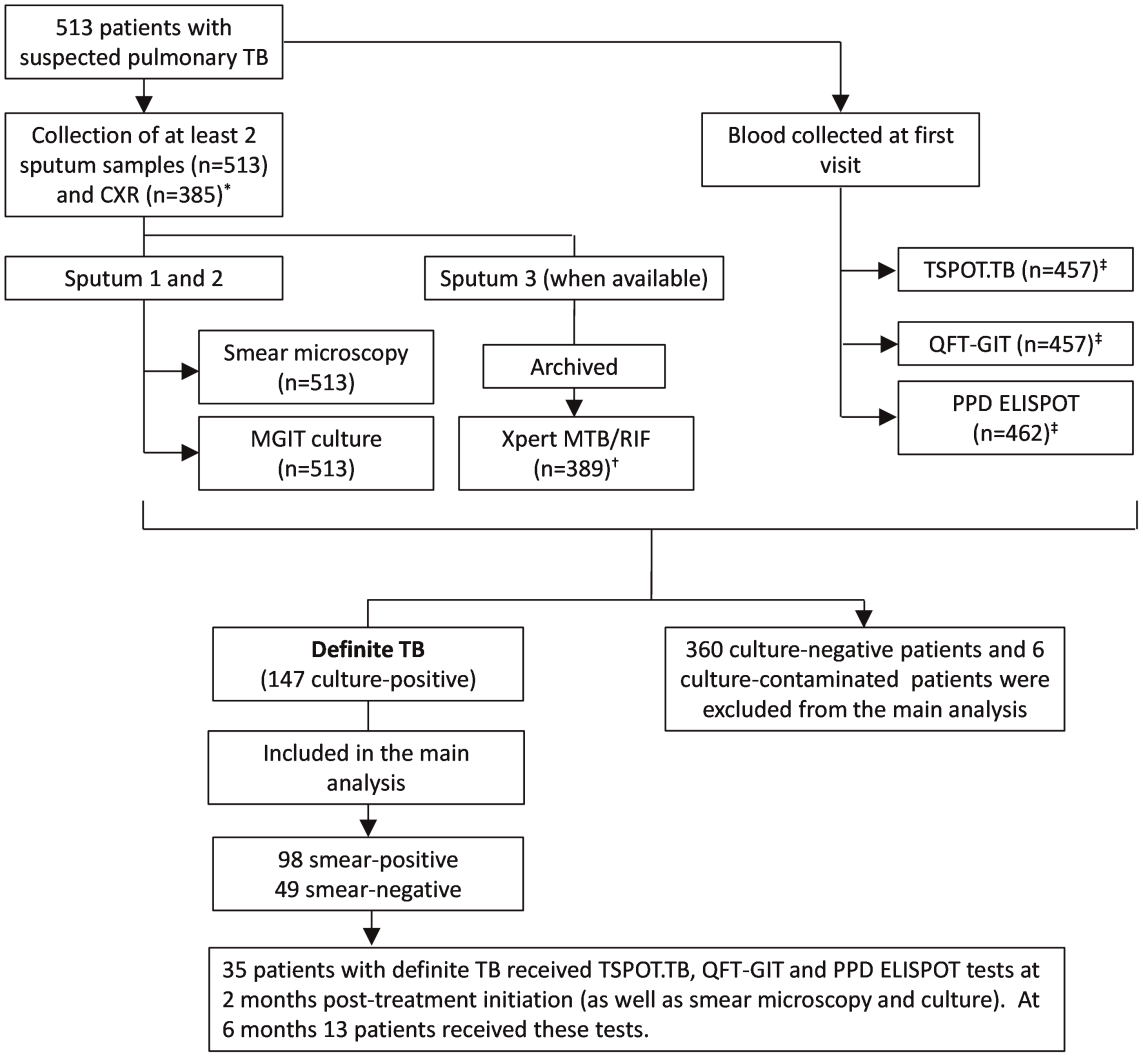
Patient flow diagram and diagnostic outcomes stratified by final diagnostic category. *128 patients had no chest X-ray data. ^†^To be eligible for the study, patients needed to provide at least 2 sputum samples (spot or morning), both of which were used for concentrated fluorescent smear microscopy and liquid culture. When available, a paired second spot sputum sample was collected and archived. This was later used for Xpert MTB/RIF testing (n = 389). ^‡^The TSOT.TB, QFT.GIT and the PPD ELISPOT tests were not all concurrently available throughout the duration of study and thus not all patients necessarily received a combination of all 3 IGRAs. The number of valid results for TSPOT.TB, QFT-GIT and PPD respectively were 457, 457, and 462, respectively. 9 TSPOT.TB indeterminate results and 52 indeterminate QFT-GIT results occurred and are excluded. Only patients with culture-confirmed TB were included in the main analysis for the comparison of IGRA-response with bacterial load.

Sputa and blood were collected at the time of diagnosis from 513 consecutively recruited ambulatory patients with suspected TB (≥18 years of age) between February 2007 and April 2010 at two primary care clinics in Cape Town, South Africa (Figure 1). Informed written consent was obtained from all participants and the study was approved by the University of Cape Town, Faculty of Health Sciences Research Ethics Committee. Detailed patient and laboratory-specific information were recorded on a standardized case record form and captured using double data entry.

### Patient Flow and Diagnostic Tests

All patients had at least one concentrated fluorescent smear and one BACTEC MGIT 960 (BD Diagnostics, USA) liquid culture performed on spot or morning sputum (Figure 1). Culture positivity for *M. tuberculosis* served as a reference standard for definite TB. An HIV test was performed after appropriate counselling. When available, a sputum sample was archived and later used for Xpert MTB/RIF (Cepheid, Sunnyvale, USA) testing [27]. Blood was collected at recruitment and used for the TSPOT.TB, QFT-GIT and PPD enzyme-linked immunosorbent spot (ELISPOT) assays. Where possible, a chest X-ray [CXR; read by 2 readers blinded to clinical information using the validated CXR scoring and recording system (CRRS) [28], [29]] was performed at recruitment. 35 randomly selected TB patients received, in addition to routine follow-up smear microscopy and culture, a TSPOT.TB, QFT-GIT and PPD ELISPOT test at 2 months post-treatment initiation. 13 of these patients received these tests again at 6 months. The accuracy of TSPOT.TB, QFT-GIT, Xpert MTB/RIF and CRRS for the diagnosis of TB in this cohort has previously been published [8], [27], [30], [31].

### Interferon-γ Release Assays

All tests were performed according to the manufacturers’ instructions on blood by laboratory technicians blinded to patient information. PBMCs were isolated from the blood and each IGRA performed within 8 hours of venepuncture. TSPOT.TB, QFT-GIT and PPD ELISPOT results were reported and interpreted as described previously [8], [32].

### Data Analysis

Culture positivity for *M. tuberculosis* from at least a single sample served as a reference standard for definite TB. Statistical analyses were performed using Graphpad Prism (version 5.0; GraphPad Software, San Diego, USA, www.graphpad.com) and OpenEpi (version 2.3.1) [33]. Statistical tests on longitudinal changes in IFN-γ levels were done on a per patient basis. TSPOT.TB and QFT-GIT results defined as indeterminate according to the manufacturers’ definitions were excluded. All data was also analysed stratified by HIV status.

## Results

### Baselines Characteristics of Study Population

Table 1 and Figure 1 show the baseline characteristics of the cohort. Of the 513 participants, 63% (354/513) were male and 26% (133/445) were HIV-infected. 29% (147/513) of participants had definite TB, 67% (98/147) of whom were smear-positive. TB patients were significantly more likely to be younger (p<0.01), of black (p<0.01) or mixed ancestry (p<0.01), have a history of previous TB (p = 0.02), HIV-infected (p<0.05) and TSPOT.TB- (p<0.01) or QFT-GIT-positive (p<0.01).

**Table 1:** Demographic and clinical characteristics of patients stratified by culture status.

Demographic or clinical characteristic	Study cohort (%) (n = 513)	Culture-positive TB (%) (n = 147)*	Culture-negative TB (%) (n = 360)*	P-value
**Age**				
Mean years (range)	39 (18–83)	37 (19–71)	39 (18–83)	<0.05
**Sex**				
Male	354 (69)	93 (63)	255 (71)	0.10
Female	159 (31)	54 (37)	105 (29)	-
**Race**				
Black	363 (71)	109 (74)	250 (69)	0.24
Mixed ancestry (coloured)	140 (27)	36 (25)	103 (29)	0.38
White	10 (2)	2 (1)	10 (2)	0.39
**Previous tuberculosis**				
Yes	172 (34)	38 (26)	132 (37)	0.02
No	321 (63)	102 (69)	215 (60)	0.04
Unknown	20 (4)	7 (5)	13 (4)	0.55
**HIV-infected**				
Yes	133 (26)	47 (32)	85 (24)	0.06
No	312 (61)	85 (58)	224 (62)	0.62
Unknown	68 (13)	10 (4)	51 (14)	0.02
**CD4 count** (cells/ml) (range)†	198 (7–935)	199 (7–894)	192 (14–935)	0.10
**TSPOT.TB**				
Positive	297 (58)	115 (78)	180 (50)	<0.01
Negative	160 (31)	18 (12)	138 (38)	<0.01
Indeterminate	9 (2)	1 (1)	8 (2)	0.26
Unknown	47 (31)	13 (9)	34 (9)	0.84
**QFT-GIT**				
Positive	311 (61)	111 (76)	196 (54)	<0.01
Negative	146 (28)	19 (13)	40 (11)	<0.01
Indeterminate	52 (10)	15 (10)	36 (10)	0.93
Unknown	4 (1)	2 (1)	2 (1)	0.41

* 6 patients did not have a useable culture result (culture contaminated) and were excluded.

† Performed if HIV-infected. Excludes 8 patients with no CD4 count data.

### Correlation of Interferon-γ Responses with Liquid Culture

**Figure 2:**
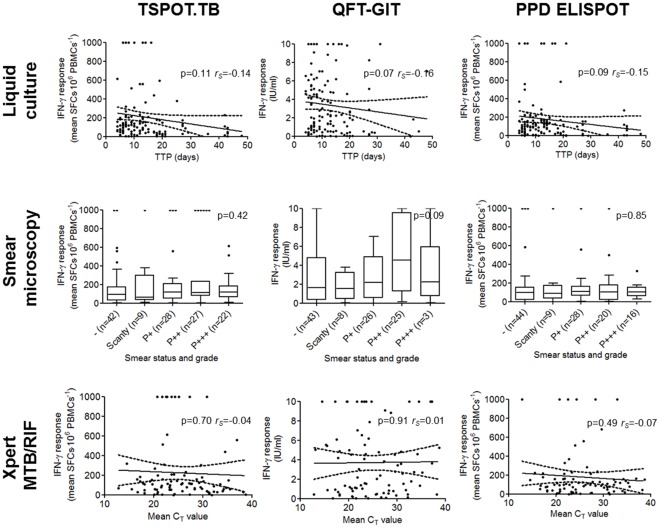
IFN-γ responses measured using the TSPOT.TB, QFT-GIT and PPD ELISPOT assays and correlated with liquid culture time-to-positivity, smear status and grade, and Xpert MTB/RIF-generated C_T_ values in individuals with culture-confirmed TB. Culture, smear-microscopy and the Xpert MTB/RIF assay were performed on a paired sputum sample collected at diagnosis. P-values and Spearman’s rank correlation coefficient (*r_S_*) are shown where appropriate. Abbreviations: SFC, spot-forming colonies; IFN-γ, interferon–γ; QFT-GIT, QuantiFERON TB Gold-in-tube; ELISPOT, enzyme-linked immunosorbent spot; PBMCs, peripheral blood mononuclear cells; IU, international units; TTP, time-to-positivity; C_T_, cycle threshold.

Median IFN-γ responses differed significantly in culture-positive vs. -negative individuals when measured using TSPOT.TB [111 (IQR: 500-194) vs. 32 (10–104) SFC⋅10^−6^ PBMCs; p<0.01] and QFT-GIT [2.2 (0.6, 5.2) vs. 0.7 (0.1, 3.1) international units (IU)⋅ml^−1^; p<0.01], but not in response to PPD [100 (36, 161) vs. 86 (16, 157) SFC⋅10^−6^ PBMCs; p = 0.28]. On average, the magnitude of IFN-γ response decreased with increasing TTP in individuals with definite TB, however, this did not reach significance [p-value; Spearman’s rank correlation coefficient (*r_S_*)] for TSPOT.TB (0.11; −0.14), QFT-GIT (0.07; −0.16) or PPD ELISPOT (0.09; −0.15) (Figure 2).

### Correlation of Interferon-γ Responses with Smear Status and Grade

Median IFN-γ responses did not differ significantly in individuals with smear-positive vs. -negative definite TB [114 (IQR: 63-208) vs. 92 (30, 172) SFC•10^−6^ PBMCs for TSPOT.TB (p = 0.12); 2.3 (0.7–5.5) vs. 1.6 (0.4–4.8) IU⋅ml^−1^ for QFT-GIT (p = 0.14); 100 (51–158) vs. 99 (18–151) SFC⋅10^−6^ PBMCs for PPD (p = 0.39)]. IFN-γ responses did not significantly correlate with increasing smear grade for either assay (p-values for T.SPOT.TB, QFT-GIT and PPD of 0.42, 0.09, and 0.85, respectively) (Figure 2).

### Correlation of Interferon-γ Responses with Xpert MTB/RIF Performance

Median IFN-γ responses differed significantly in Xpert MTB/RIF-positive (130/480) vs. Xpert MTB/RIF-negative (350/480) individuals when measured using the TSPOT.TB [109 (IQR: 49–206) vs. 40 (10–115) SFC⋅10^−6^ PBMCs; p<0.01] and QFT-GIT assays [2.2 (0.6–5.4) vs. 0.7 (0.1–3.1) IU⋅ml^−1^; p<0.01], but not in response to PPD [100 (45–160) vs. 91 (17–185) SFC⋅10^−6^ PBMCs; p = 0.21]. On average, IFN-γ responses decreased with increasing Xpert MTB/RIF-generated cycle threshold (C_T_) values in Xpert MTB/RIF-positive individuals with definite TB, however, this relationship was not significant (p-value; *r_S_*) for TSPOT.TB (0.70; −0.04), QFT-GIT (0.91; 0.01) or PPD ELISPOT (0.49; −0.07) (Figure 2).

### Correlation of Interferon-γ Responses with Chest Radiography Data

IFN-γ responses differed significantly in individuals who had CXR suspicious of active TB (344/416) vs. those who had a normal CXR (72/416), when measured using TSPOT.TB [58 (IQR: 15–153) vs. 31 (8–101) SFC⋅10^−6^ PBMCs; p<0.01] and QFT-GIT assays [1.1 (0.2–4.6) vs. 0.3 (0.0–2.3) IU⋅ml^−1^; p<0.01], but not in response to PPD [96 (28–180) vs. 92 (20–142) SFC⋅10^−6^ PBMCs; p = 0.37]. In patients with definite TB who had a CXR compatible with active disease, median IFN-γ responses did not differ significantly between those who had cavities (52/124) and those who did not (72/124) when measured using TSPOT.TB [140 (80–208) vs. 112 (33–190) SFC⋅10^−6^ PBMCs; p = 0.12], QFT-GIT [3.0 (1.0–5.8) vs. 1.6 (0.4–4.6) IU⋅ml^−1^; p>0.05] or PPD ELISPOT [106 (28–194) vs. 117 (34–159) SFC⋅10^−6^ PBMCs; p = 0.88].

### Impact of HIV-status on Interferon-γ Response

HIV-infected patients with definite TB had a similar IFN-γ response compared to those without HIV-infection when measured using the TSPOT.TB assay [86 (IQR: 31–158) vs. 115 (56–200); p = 0.22], but when measured using QFT-GIT [2.2 (0.4–3.8) vs. 3.7 (0.9–6.1); p = 0.02] or the PPD ELISPOT [62 (20–118) vs. 122 (62–196); p<0.01] the magnitude of response was significantly lower in the HIV-infected group. When the definite TB group was stratified by HIV-status, the correlation between IFN-γ response and bacterial load proxy [for each marker (including cavitation) and for each IGRA] was non-significant, irrespective of HIV-status (data not shown).

### Smear- and Culture-status of Patients Undergoing Treatment

The status of the 35 culture-positive patients who were available for IGRA-based follow-up is shown in Table 2. After 2 months of anti-TB therapy, 60% (21/35) of these individuals had culture-converted and were identified as responders to therapy.

**Table 2:** Status of TB patients at diagnosis (all culture-positive) and after 2 and 6 months of anti-TB treatment.

	Months of anti-TB treatment		
	0 months (n = 35)	2 months (n = 35)	6 months (n = 13)
**Converters (%)** (from positive at 0 months to negative at 2 or 6 months)			
Smear microscopy*	N/A	63 (15/24)	100 (9/9)
Liquid culture	N/A	60 (21/35)	77 (10/13)
TSPOT.TB†	N/A	11 (3/28)	8 (1/13)
QFT-GIT‡	N/A	5 (1/29)	30 (3/11)
**Median IFN-γ response** § (IQR)			
TSPOT.TB (SFC⋅10^−6^ PBMCs)	94 (35–158)	118 (57–174) (p = 0.51)	111 (50–310) (p = 0.83)
QFT-GIT (IU⋅ml^−1^)	2.8 (0.6–4.9)	2.5 (0.2–7.2) (p = 0.88)	0.9 (0.0–7.6) (p = 0.32)
PPD (SFC⋅10^−6^ PBMCs)	100 (30–153)	90 (46–127) (p = 0.26)	136 (96–309) (p = 0.11)

* Of the 24/35 individuals who were smear-positive at 0 months, 15/24 had converted at 2 months. Of 13 individuals followed up at 6 months, 9 were smear-positive at month 0 and all 9 had converted at 6 months.

† Of the 28/35 individuals who were TSPOT.TB-positive at 0 months, 3/28 had converted at 2 months. All 13 individuals at month 6 were TSPOT.TB-positive month 0.

‡ Of the 29/35 individuals who were QFT-GIT-positive at 0 months, 1/29 had converted at 2 months. 11 of the 13 individuals followed up at month 6 were QFT-GIT-positive at month 0. 2/35 individuals had an indeterminate QFT-GIT result at month 0.

§ Per patient p-values are shown for comparisons of IFN each IGRA for either 2 or 6 months vs. 0 months.

Abbreviations: SFC, spot forming colonies; PBMCs, peripheral blood mononuclear cells; IU, international units.

### Longitudinal Changes in Interferon-γ Release Assay Status

11% (3/28) of individuals who were TSPOT.TB-positive at 0 months were negative at 2 months and 5% (1/29) of individuals with TB who were QFT-GIT-positive at 0 months were negative at 2 months (Table 2). In contrast, 63% (15/24) of smear-positive individuals at 0 months were negative at 2 months (p-values of <0.01 and <0.01 compared to TSPOT.TB and QFT-GIT, respectively). 60% (21/35) of culture-positive individuals at 0 months were negative at 2 months (p-values of <0.01 and <0.01 compared to TSPOT.TB and QFT-GIT, respectively). At 6 months, the proportions of patients who had converted were 8% (1/13) and 30% (3/11) for TSPOT.TB and QFT-GIT respectively.

### Longitudinal Changes in Quantitative Interferon-γ Responses

**Figure 3:**
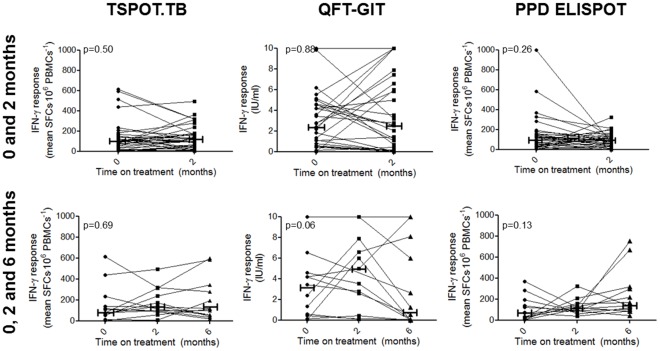
Longitudinal IFN-γ responses are shown for patients undergoing anti-TB treatment at 2 months (n = 35) and 6 months (n = 13). Capped horizontal bars represent the median IFN-γ response at each time-point.

Quantitative IFN-γ responses to RD-1 specific antigens or PPD did not change on a per patient basis between 0 vs. 2 months (p-values of 0.51, 0.88 and 0.26 for TSPOT.TB, QFT-GIT and PPD, respectively) or between 0 vs. 6 months (p-values of 0.83, 0.32, and 0.11 for each test) in individuals with definite TB (Figure 3). There was also no significant longitudinal change in per patient IFN-γ levels across all 3 time points (p-values of 0.69, 0.06 and 0.13 for each test respectively). Furthermore, no longitudinal changes in IFN-γ response were detected in individuals who culture converted at 2 months, irrespective of the type of IGRA used (p-values of 0.27, 0.60, and 0.22 respectively for individuals culture-negative at 2 months; p-values of 0.08, 0.24 and 1.00 for TSPOT.TB, QFT-GIT and PPD respectively for individuals still culture-positive at 2 months) (Table 3). IFN-γ responses also did not differ in culture-negative vs. –positive individuals at this time point [96 (IQR: 24-164) vs. 136 (77 vs. 210) SFC⋅10^−6^ PBMCs for TSPOT.TB (p = 0.21), 3.2 (0.0–8.9) vs. 1.3 (0.3–4.4) for QFT-GIT (p = 0.78), and 93 (50–125) vs. 96 (51–166) for PPD (p = 0.67)] nor were there any differences in baseline IFN-γ response between 2 month culture converters and non-converters [110 (34–207) vs. 76 (4 vs. 98) SFC⋅10^−6^ PBMCs for TSPOT.TB (p = 0.10), 2.2 (0.4–4.9) vs. 4.2 (0.5–5.3) for QFT-GIT (p = 0.63), and 122 (27–161) vs. 81 (27–157) for PPD (p = 0.57)].

**Table 3:** Differences in IFN-γ responses between culture converters (positive-to-negative) and non-converters after 2 months of anti-TB treatment.

	2 month culture status (n = 35)					
	Culture-negative (n = 21)			Culture-positive (n = 14)		
	Median IFN-γ response (IQR) at 0 months	Median IFN-γ response (IQR) at 2 months	P-value*	Median IFN-γ response (IQR) at 0 months	Median IFN-γ response (IQR) at 2 months	P-value*
**TSPOT.TB** (SFC⋅10^−6^ PBMCs)	110 (34–207)	96 (24–164)	0.27	76 (4–98)	136 (77–210)	0.08
**QFT-GIT** (IU⋅ml^−1^)	2.2 (0.4–4.9)	3.2 (0.0–8.9)	0.60	4.2 (0.6–5.0)	1.3 (0.2–3.9)	0.24
**PPD** (SFC⋅10^−6^ PBMCs)	122 (27–161)	93 (50–125)	0.22	81 (27–157)	96 (51–166)	1.00

* Per patient P-values are shown for each IGRA for comparisons between 0 and 2 months.

At 6 months post-treatment initiation IFN-γ levels did not change significantly from diagnosis in individuals who had culture-converted (p-values of 1.00, 0.08 and 0.46 for TSPOT.TB, QFT-GIT and PPD respectively). Due to the small number of culture-positive TB cases after 6 months of treatment (n = 3), we were unable to conclusively compare differences in IFN-γ responses between converters and non-converters at this time point, however, all 3 6 month non-converters were TSPOT.TB- and QFT-GIT-positive.

## Discussion

It has been suggested that IFN-γ responses to RD1-specific and non-specific antigens may be useful as prognostic tools, and for monitoring bacterial load and treatment response [2]. However, there is limited information from high-burden settings. Our main aim was to evaluate, at diagnosis, the relationship between IGRAs and measures of bacterial load, which is a prognostic tool associated with treatment failure [1], in patients with TB. We further comparatively evaluated one in-house and two standardised IGRAs longitudinally in relation to 2-month culture conversion, a measure known to be associated with treatment-related outcome [1].

The key findings of our study were: (i) T-cell IFN-γ responses do not correlate with measures of bacterial load in TB patients at diagnosis including culture TTP, smear status and grade, Xpert MTB/RIF C_T_ values, or the presence of cavities on the CXR and this occurred irrespective of HIV status; (ii) IFN-γ levels (whether in response to the RD1-specific antigens or PPD) do not change significantly after 2 or 6 months of anti-TB treatment, irrespective of culture conversion status.

We did not detect any relationship between quantitative IFN-γ responses and bacillary load at diagnosis irrespective of HIV status. By contrast, a smaller study in HIV-infected TB patients in similar setting found a significant but weak correlation between quantitative TSPOT.TB responses and smear grade (a relatively crude measure of bacterial load) but none with culture TTP [10]. Cavitation on the radiograph, previously shown to be associated with either significantly elevated sputum bacillary load or RD1-specific responses in low burden settings [21], [34], was not associated with elevated IFN-γ responses in our study. There is, to the best of our knowledge, no published literature on the correlation of IFN-γ responses with Xpert MTB/RIF C_T_ values. The poor relationship between IFN-γ response and bacillary load may be a function of T cell compartmentalisation and immunosuppressive mediators including IL-10, TGF-β and regulatory T cells [35], [36]. Thus, TB-specific T cells are concentrated in the lung and not in the sampled compartment, and those with the most extensive disease may have the greatest attenuation of RD-1-specific Th1 immunity. Whether other antigens such as HBHA behave differently remains unclear [37]–[40].

Are serial IFN-γ responses useful for monitoring patient-related outcomes in TB patients undergoing treatment? A comparative analysis of the different IGRA formats for treatment monitoring (or correlation of initial bacillary load) has not previously been undertaken. The majority of studies that have examined longitudinal IGRA responses have been performed in a low burden setting [17], [19], [23], [24]. In our study, and similar to findings in The Gambia where an in-house assay was used [25], standardised IFN-γ responses to both RD1-specific antigens and PPD did not appreciably change longitudinally. Although the sensitivity of the T-SPOT and QFT-GIT assays are known to differ [6] we found no differences in the longitudinal readouts. In contrast to a previous study which found that RD-1, but not PPD responses, selectively diminished during the course of treatment [16] we found that responses to both antigens remained persistently elevated. Of note, however, is that this earlier study used an in-house experimental assay which, in contrast to the commercially available TSPOT.TB or QFT-GIT tests, utilised two ESAT-6-derived peptides. This, rather than setting-specific differences with our study, might account for these discordant results, especially given that the findings of the original study have since been confirmed in India, where a similar assay was used [41].

Given the poor correlation we found between IFN-γ responses and culture TTP and cavitation at diagnosis, both of which are known associates of 2-month culture conversion [34], [42]–[45]; it is unsurprising that we also detected no association between IFN-γ responses and this outcome. This high proportion of persistent T-cell responses may be due to several factors including: a history of repeated or on-going TB exposure in a high burden environment, the slow decay kinetics of T cells [24], [46], or greater T cell stimulation with antigen release during the course of treatment. Indeed, history of past-exposure is likely to cause an initially high IFN-γ response which, in patients with latent TB, has shown to remain largely unchanged throughout chemotherapy [46].

There are several limitations to this study. Firstly, all patients did not receive the same combination of tests due to the study design. Although this restricted our sample size, our study nonetheless remains the largest that has specifically looked at the issue of IFN-γ responses and bacillary load. Second, only a subset of patients underwent 6 month follow-up and, as a result, the conclusiveness of our findings limited. Third, given the relatively high prevalence of TB and HIV co-infection in South Africa, these findings may not be generalizable to other settings. Fourth, the Xpert MTB/RIF assay in this cohort was performed on archived sputum samples, which may influence the C_T_ values generated by the assay, however, this effect has previously not shown to be significant [27], [47]. Finally, it is worth noting that culture conversion itself correlates only crudely with unfavourable treatment outcomes [1] but nevertheless remains the best surrogate biomarker of treatment outcome.

In conclusion, our data suggest that T-cell IFN-γ responses to either RD1-specific or non-specific antigens do not correlate with bacillary burden or treatment outcome amongst TB patients in a high burden setting. They are hence unlikely to have utility as prognostic tools at initial evaluation or to monitor either disease severity or treatment response.
